# Evaluating the Efficacy of Marma Therapy for Pain in Lumbar Disc Herniation With Radiculopathy: Protocol for a Randomized Controlled Trial

**DOI:** 10.2196/85720

**Published:** 2026-05-04

**Authors:** Pallavi Mundada, Sunil Kumar Joshi, Shruti Khanduri, Tarun Kumar, Varsha Saxena, Disha Garg, Vinod Kumar Lavaniya, Rakesh Narayanan V, Bidhan Mahajon, Bhogavalli Chandra Sekhara Rao, Narayanam Srikanth

**Affiliations:** 1 Central Council for Research in Ayurvedic Science, New Delhi, Delhi, India; 2 Faculty of Ayurveda, Uttarakhand Ayurved University, Dehradun, Uttarakhand, India; 3 Regional Ayurveda Research Institute, Central Council for Research in Ayurvedic Science, Village Thapla P.O. Box 263645, Ganiyadholi, Ranikhet, Almora, Uttarakhand, India, 91 8851724923

**Keywords:** Ayurveda, lumbar disc herniation, marma therapy, radiculopathy, low-back pain

## Abstract

**Background:**

*Marma* therapy, a traditional Ayurvedic practice, involves the precise stimulation of *marmas* (vital points that regulate *prana* [vital energy]) and alleviates musculoskeletal pain and dysfunction. While historical texts describe *marma*’s role in pain relief, no randomized controlled trials have evaluated its efficacy and safety in lumbar disc herniation (LDH)–related radiculopathy.

**Objective:**

This study aims to explore the efficacy and safety of *marma* therapy in a commonly occurring painful condition, namely, radiculopathy due to LDH.

**Methods:**

Selected patients with LDH and radiculopathy are randomized into 2 groups using a computer-generated random number sequence. The participants in group 1 (the trial arm) are treated with *marma* therapy for 4 weeks, and those in group 2 (the control arm) receive physiotherapy for 4 weeks. All the participants in both groups are given an oral medicine, *trayodashanga guggulu*, an Ayurvedic formulation, for 12 weeks.

**Results:**

As of November 2025, a total of 90 patients have been enrolled in both groups. Data analysis is ongoing. The study will be reported following standard guidelines for reporting randomized controlled trials. Clinical trial results will be disseminated through conferences and publication in a peer-reviewed scientific journal.

**Conclusions:**

*Marma* therapy, if proven effective and safe in pain management, can improve the quality of life of patients with LDH. This protocol can be useful in designing large-scale studies to establish this noninvasive and safe treatment as an alternative modality for the management of neurological pain, such as radiculopathy.

## Introduction

Low-back pain (LBP) with radiculopathy due to lumbar disc herniation (LDH) is a leading cause of disability worldwide, affecting approximately 5% to 10% of patients with chronic LBP [1,2], with up to a 70% lifetime prevalence [3]. Radiculopathy, characterized by radiating pain, sensory deficits, and motor weakness along the affected nerve root, significantly impairs the quality of life (QoL) and functional capacity [4]. Conventional treatments, including analgesics, physical therapy, epidural steroid injections, and surgery, often provide incomplete relief and carry risks of adverse effects, recurrence, and high costs [5].

Given these challenges, complementary and alternative medicine approaches, including Ayurveda, are increasingly explored for neuropathic pain management. Existing research on Ayurvedic therapies for LBP and radicular pain suggests potential benefits. However, the evidence is preliminary and primarily based on case reports. Only a few randomized controlled trials (RCTs) on Ayurvedic procedures for LBP have been published. One of them shows promising results for *Abhyanga* (Ayurvedic oil massage) in chronic LBP [6]. *Marma* therapy, an Ayurvedic intervention, involves stimulating vital energy points (*marma* points) to restore physiological balance and alleviate pain. While *marma* therapy is traditionally used in Ayurveda for musculoskeletal dysfunctions [7], its efficacy in LDH-associated radiculopathy remains understudied in rigorous clinical trials. Studies on yoga and acupuncture, both of which share conceptual similarities with *marma* therapy in terms of energy modulation, have demonstrated efficacy in LBP [8]. However, no RCT has specifically evaluated *marma* therapy for LDH-associated radiculopathy.

This study aims to assess the efficacy of *marma* therapy in pain management and functional improvement in patients with LDH-associated radiculopathy, compared with standard physiotherapy. The findings will contribute to evidence-based integrative approaches for neuropathic pain management. This is the first RCT that evaluates the efficacy and safety of *marma* therapy in LDH-associated radiculopathy compared to standard physiotherapy. The primary objective of this study is to evaluate the efficacy of *marma* therapy in reducing pain associated with lumbar radiculopathy caused by intervertebral disc herniation. Outcomes include pain intensity, measured by the Visual Analog Scale (VAS); functional disability, assessed by the Oswestry Disability Index (ODI); and QoL, evaluated by the 36-item short form health survey (SF-36). This study also assesses the safety of *marma* therapy among the selected patients. The study aims to fill a critical evidence gap in integrative pain management.

## Methods

### Participants, Interventions, and Outcomes

#### Study Design

This is a randomized, open-label, parallel-arm, active-controlled clinical trial.

#### Study Setting

The study is conducted at the outpatient department (OPD) of the Main Campus Hospital, Uttarakhand Ayurved University, Dehradun, Uttarakhand, India.

### Eligibility Criteria

#### Inclusion Criteria

Participants of any sex, aged 30-60 years, diagnosed with lumbar radiculopathy with unilateral radiating pain (either left or right lower limb) due to intervertebral disc herniation confirmed by magnetic resonance imaging (MRI; disc herniation between L3 and S1 levels at a single or double level) and without any evident neurological deficit are included. Participants having a positive straight leg raise test (SLRT; ipsilateral or contralateral pain in the leg, buttock, or back at ≤70° of leg elevation, with pain typically worsened by dorsiflexion of the ankle or neck flexion after slowly lowering the leg until the patient no longer feels pain) and those who are willing to adhere to the study protocol for a period of 3 months are included in this study.

#### Exclusion Criteria

Patients who are indicated for surgical intervention for disc herniation (eg, those with severe motor deficit [motor power of the lower limbs assessed through the Medical Research Council Manual Muscle Testing scale ≤3] or severe spinal stenosis); those with excruciating pain that cannot be managed by conservative treatment; or those with evident foraminal stenosis, conjoint nerve root, or perineural cyst are excluded. Patients who have received nonpharmacological interventions, such as physiotherapy, traction, or manual therapy, for the management of LDH in the last 3 months are also excluded. Patients having a history or evidence of osteoporotic lumbar fracture, spinal trauma, spinal malignancy, or inflammatory or infective diseases that affect spinal morphology (such as ankylosing spondylitis, spondylodiscitis or inflammatory spondylitis, spondylolisthesis, Pott disease, piriformis syndrome, or sacroiliitis); a history of spinal surgery in the last 2 years; or epidural fibrosis are excluded. Patients with cauda equina syndrome or neurological deficits, such as foot drop, limb muscle wasting, or bowel or bladder incontinence or nonambulatory patients with monoplegia, paraplegia, or hemiplegia are also excluded. The presence of other medical conditions presenting numbness and pain in the lower extremities (such as diabetic polyneuropathy, peripheral vascular disease, motor neuron disease, multiple sclerosis, or stroke), cognitive impairment, or major coexisting medical conditions such as cancer, chronic obstructive pulmonary disease, cardiovascular disease, or severe hepatic or renal dysfunction is also an exclusion criterion. Patients with obesity (BMI ≥30 kg/m^2^), those not suitable for MRI (eg, those with metallic implants such as pacemakers or hearing aid implants), or those having any other condition that limits the participation of the patient, as determined by the investigator, are also excluded from the study.

### Interventions

The participants were randomly allocated to 1 of 2 groups. Both groups receive a different nonpharmacological therapy along with a common Ayurvedic formulation for oral consumption. Group 1 received *marma* therapy for 4 weeks and *trayodashanga guggulu* for 12 weeks, and group 2 received physiotherapy for 4 weeks along with *trayodashanga guggulu* for 12 weeks.

*Marma* therapy is a procedure that includes stimulation of *kshipra* (point located between the great toe and the second digit of the lower limb)*, gulpha* (point located posterior to the medial malleolus)*, indrabasti* (point located 8 fingerbreadths above the first skin fold of the ankle joint)*, katikataruna* (points located bilaterally along the vertebral column over the hip bone), and *janu* (point located posterior to the knee joint) *marma* of the affected limb thrice daily. Out of 108 *marma* points mentioned in the Ayurvedic texts, these were selected for the said condition, based on the clinical experience of the principal investigator.

*Trayodashanga guggulu* is a classical multiherb Ayurvedic formulation mentioned in the Ayurvedic Formulary of India, Part-1 [9]. This medicine is procured from a Good Manufacturing Practice–certified pharmacy, and its quality is in compliance with the standards mentioned in the Ayurvedic Pharmacopoeia of India [10].

### Delivery of Interventions

The mode of delivery, frequency, and duration of each intervention are described below.

For *marma* therapy, the participants in the trial arm are advised to wear loose and comfortable clothing (without ties, belts, stockings, or such binding clothes) and lie down in the prone or supine position, as per the site to be stimulated. Each of the 5 *marma* points is stimulated in an upward-to-downward direction in each session. *Marma* points are stimulated by gentle pressure or vibration using the pulp of the thumb or fingers and the inner side of the knuckle. Each session includes 18 to 20 stimulations to each of the said *marma* points (1 stimulation lasts for 0.8 seconds). Three such sessions are done each day for 4 weeks. *Marma* stimulation in female patients is typically performed first in the left extremity, followed by the right extremity, and vice versa in male patients.

For physiotherapy—educational training (eg, advice to avoid sitting for long periods, forward bending, lifting heavy object from ground using leg with knee bending, and trying to tighten the stomach muscles) along with electrotherapy (transcutaneous electrical nerve stimulation with frequency of 20 Hz; pulse width of 100 µs; continuous mode; and 2 channels for 15 minutes, with electrodes placed along the lines of the T12 and S1 vertebrae and intensity increased slowly to a tolerably painful level); hot pack therapy (water at a temperature of 54 °C placed at the lower back for at least 15 minutes, with the patient in hook-lying position); therapeutic exercises suggested based on the case (mostly including drawing-in maneuver, multifidus activation and training, cat-camel exercise, arm lifts and bracing, leg lifts and bracing, single knee-to-chest exercise, and exercises to promote mobility, strength, and range of motion in the lower back); and manual therapy (flexibility training) were provided for 30 minutes per session, 5 sessions per week, for 4 weeks. The participants in the control arm are instructed on the therapeutic exercises and provided with a chart outlining the final positions and necessary instructions.

*Trayodashanga guggulu* tablets, weighing 500 mg each, are given as oral medication to the study participants in both groups at a dose of 1 g thrice daily, 2 hours after meals, with lukewarm water for 3 months. Participants are allowed to take oral analgesics as rescue medication for pain relief upon telephonic consultation with the investigator. Ongoing medications reported at baseline for any chronic condition (not mentioned in the exclusion criteria) are also continued by the study participants as concomitant medication. Participants who are unwilling to continue, are noncompliant with the study procedure, or meet any exclusion criteria during the study period are withdrawn from the study.

### Outcome Measures

The primary outcome of this study is a reduction in pain intensity in the lumbosacral region and legs (radiating pain) perceived by participants, assessed as the change in the 10 cm VAS score at day 30 and after 3 months from baseline.

The secondary outcomes of this study are improvement in functional disability due to LDH, assessed by the ODI, version 2.0; change in neurodynamic evaluation, assessed using the SLRT; improvement in QoL, assessed by the SF-36; comparison of dependence on allopathic analgesics; and assessment of safety and acceptability of the *marma* therapy in patients with LDH, assessed by the incidence of adverse events (AEs) associated with the therapy and compliance of patients to *marma* therapy.

### Participant Timeline

Eligible participants, upon signing the informed consent form, are assessed for screening according to the criteria outlined in the study protocol. Spine examination and neurological assessment by a spine surgeon, systemic examination, SLRT, MRI of the lumbosacral spine, and laboratory investigations (including hemogram, liver function test, renal function test, C-reactive protein, hemoglobin A1c, random blood sugar, rheumatoid factor, and human leukocyte antigen B27 test) are done during the screening of participants before baseline evaluation. Patients undergoing any internal or external therapy or Ayurvedic medication for pain management are instructed to discontinue these treatments, and a baseline evaluation is conducted after a 1-week washout period.

At the baseline visit, general information for personal identification and demographic profiling is collected, medical history is recorded, and a clinical examination for general physical and systemic conditions is done, followed by a detailed neurological examination. Assessment of pain by the 10 cm VAS, functional disability by the ODI, and QoL using the SF-36 is done. The history of consumption of nonsteroidal anti-inflammatory drugs (NSAIDs) or analgesic medications during the last 14 days is also noted. The Ayurvedic parameters, such as *prakriti*, are also assessed at baseline.

The participants are assigned the treatment as per group allocation mentioned on the chit in the opaque sealed envelopes opened by the participant upon baseline evaluation. Each study participant is called daily for the assigned therapy (*marma* therapy or physiotherapy) for 4 weeks. Oral medication has continued for 12 weeks.

Follow-up and outcome assessments are conducted on days 7, 15, 21, 30, 45, 60, and 90. Any AE reported by a study participant is recorded at each visit and thoroughly assessed for causality. Ongoing events are managed through appropriate interventions, including referral to a higher center when necessary. A telephonic follow-up on day 75 is done to note any AEs, the need for rescue medication, and drug compliance. Details of assessments done during each visit of the study participant are given in Table 1.

**Table 1.** Participant timeline.

Variable	Study period									
	Enrollment	Allocation	After allocation							
	Screening	Baseline	Day 7	Day 15	Day 21	Day 30	Day 45	Day 60	Day 75 (telephonic)	Day 90
Enrollment										
Information and written consent	✓									
Eligibility evaluation	✓									
Allocation		✓								
Intervention										
Issue of oral medication to both groups		✓	✓	✓	✓	✓	✓	✓		
Administration of *Marma* therapy to group A and physiotherapy to group B		✓	✓	✓	✓	✓				
Outcome assessment										
Medical history and demographic profile		✓								
Clinical examination	✓	✓	✓	✓	✓	✓	✓	✓		✓
Participants’ categorization into acute, subacute, and chronic cases for both groups		✓								
Baseline characteristics		✓								
Pain assessment		✓	✓	✓	✓	✓	✓	✓	✓	✓
Functional disability and quality-of-life assessment		✓		✓		✓		✓		✓
Straight leg raise test and other maneuvers		✓		✓		✓		✓		✓
Laboratory and radiological investigations	✓									✓^a^
Ayurvedic parameter assessment	✓									✓
Drug or therapy compliance			✓	✓	✓	✓	✓	✓	✓	✓
Rescue medication			✓	✓	✓	✓	✓	✓	✓	✓
Adverse event recording			✓	✓	✓	✓	✓	✓	✓	✓

a Only magnetic resonance imaging was done.

### Sample Size

A previous study that included physiotherapy as an intervention reported a mean VAS score for back pain of 3.05 (SD 1.36) points after treatment [11]. This was considered to anticipate an improvement of at least 2.79 points after *marma* therapy. Assuming a between-group difference of 1 point, with 80% power at 5% level of significance, the derived sample size was 72 participants. Furthermore, assuming a 20% attrition rate, the final sample size for this study was 90 participants, randomly allocated to the 2 groups in a 1:1 ratio (n=45, 50% participants per group). The effect size will be determined after the data analysis is completed.

### Recruitment and Participant Flow

After receiving approval from the Institutional Ethics Committee (IEC) and registering the study with the Clinical Trial Registry of India, all medical and paramedical staff are sensitized to the trial and its eligibility criteria. Patients presenting with concerns related to LBP and associated radiating pain in the lower limb in the OPDs of various departments of the hospital are referred to a special OPD for thorough assessment. Participants are recruited for the study based on the defined inclusion and exclusion criteria. A medical professional and a physiotherapist are delegated the responsibility to screen the patients clinically and get the tests conducted for the patients willing to participate and found to be eligible. Sensitization camps are conducted in nearby areas to create awareness regarding the project at the study site.

### Assignment of Interventions

The participants are randomized to 2 study groups in the ratio of 1:1. SPSS (version 15.0; IBM Corp) is used to generate the random number sequence. Allocation concealment is ensured by using the sequentially numbered, opaque, sealed envelope technique. The sequence of randomization and preparation of the sealed envelope technique is done centrally by the biostatistics unit at the sponsor’s headquarters. The envelopes are opened by the study participants after completion of the baseline assessment, and the intervention is given to them as per the group allocation. Both the participant and the investigator are aware of the group allocation at the end of the baseline assessment. This trial is an open-label study. However, the research personnel assessing the primary outcome and the secondary outcomes, namely, functional disability and neurodynamic evaluation, were not aware of the group to which participants were allocated.

### Data Collection and Management

After screening patients, the eligible participants are included in the study, and data are collected in a case record form (CRF). The data are subsequently recorded as an electronic CRF (e-CRF) on the same day. The CRF is prepared with appropriate coding to be entered into the e-CRF. Periodic data cleaning is performed to ensure the accuracy of the data entered in the e-CRF. The CRFs are stored in a secure location with restricted access.

Before initiating the study, the project team, including the investigators, is trained by the sponsor regarding the study protocol, standard operating procedures for data collection, various assessments, and Good Clinical Practice guidelines. The study participants are informed in detail about the study procedures and follow-up timelines before screening, as well as at the end of the baseline period, before the intervention begins, to confirm their consent for participation in the trial. To minimize loss to follow-up, the study team sends reminder calls and messages to participants 1 to 2 days prior to their scheduled visits.

### Data Analysis

After verification for accuracy, all collected data will be anonymized prior to statistical analysis. All analyses will follow both the intention-to-treat and per-protocol principles to evaluate the robustness and sensitivity of the results. Any missing or incomplete responses will be addressed through Bayesian imputation. To make the imputation robust, regression-based imputation will also be applied if sufficient information is available in the data. Furthermore, the data will be used for descriptive analysis. Categorical variables will be described as frequencies and percentages, while continuous variables will be summarized as mean (SD) or median (IQR), depending on the distribution of the data (normal or skewed). Inferential analyses will be performed after descriptive analysis of the data. For within-group comparisons, data will be analyzed using the paired 2-tailed t test or the Wilcoxon signed-rank test, depending on the distribution of the data for pre-post observations. Between-group comparisons will be performed using the independent-samples *t* test or Mann-Whitney *U* test, depending on the normality of the data.

Repeated-measures ANOVA or the Friedman test will be used for within-group and between-group analyses of the outcomes assessed at multiple time points. The generalized estimating equation and the repeated-measures ANOVA, along with factor analysis, will also be performed if any confounding factors are found during the analysis. Analysis of covariance will be performed if baseline differences between groups are observed. A 2-sided significance level of 5% will be considered for all analyses. All statistical analyses will be performed using Stata software (version 16.1; StataCorp).

### Monitoring

A team of technical personnel from the sponsor organization monitors the progress of the trial through regular site visits and data monitoring. The project activities at the study site are supervised to ensure compliance with the standard operating procedures outlined in the protocol and Good Clinical Practice guidelines.

The AEs reported by the study participants on the telephone or during follow-up visits are recorded in a standard format, and adequate details are collected by the study team for investigating causality. Serious AEs will be reported to the IEC and the sponsor within the stipulated timelines.

### Ethical Considerations

This research protocol has been reviewed and approved by the IEC of Uttarakhand Ayurved University, Dehradun, Uttarakhand (UAU02/30092021/M1; dated September 30, 2021). The trial is conducted in compliance with the approved protocol and the Declaration of Helsinki.

After thoroughly explaining the study to the patients and giving them the participant information sheet, written consent is obtained before screening. The signature of a witness or a legally authorized representative is obtained in cases where participants are illiterate. One copy of the signed consent form is provided to the study participant, along with the participant information sheet, for their records.

The paper source documents related to the study participants are stored in a place with restricted access, and the computer used for data entry is also password protected. All records that contain names or other personal identifiers, such as locator forms and informed consent forms, are stored separately from study records identified by code numbers. Forms, lists, logbooks, appointment books, and any other listings that link participant ID numbers to other identifying information are stored in a separate, locked file in a restricted area.

The data are analyzed after anonymization. The data will be identified only by coded ID numbers to maintain participant confidentiality. The data will be accessible to the investigators and sponsors. The IEC or any other regulatory body may also be able to access the data after appropriate procedures.

Participants will receive nominal compensation for loss of wages and conveyance costs, with ₹100 (approximately US $1.19) provided for each hospital visit during the study period.

### Dissemination Policy

The dissemination of results will be done after the completion of the study and data analysis. The results will be published without disclosing the personal IDs of the participants.

### Ancillary and Posttrial Care

No ancillary studies are proposed for this trial. The participants receive routine Ayurvedic medical care, if required, after completion of the study period.

### Protocol Amendments

Deviations from the protocol will not be made except when necessary to alleviate an immediate hazard to trial patients. All protocol amendments, including changes to interventions, examinations, data collection, and methods of analysis, will be reported to the sponsors and the IEC at the earliest opportunity, along with the exact reasons.

## Results

Participant recruitment was initiated on April 4, 2022, and as of November 2025, a total of 90 patients have been enrolled. Of these, 70 (77.8%) study participants have completed the final follow-up. Data cleaning is now complete, and the results will be published once the analysis is complete. The final report and data interpretation are expected to be submitted for publication in 2026.

## Discussion

Degenerative disease of the lumbar spine is a significant cause of disability worldwide; it encompasses conditions such as spondylolisthesis, disc degeneration, and lumbar spinal stenosis. Associated with a variety of clinical symptoms, including lower extremity pain, weakness, and LBP of varying levels of severity, lumbar degenerative spine disease can lead to a reduction in QoL [12]. A systematic review on the efficacy of various treatment modalities for herniated lumbar discs showed that the highest prevalence is among people aged 30 to 50 years, with a male-to-female ratio of 2:1. There is little evidence to suggest that drug treatments are effective in treating herniated discs. Neither NSAIDs, cytokine inhibitors, muscle relaxants, nor epidural corticosteroid injections were found to be effective in improving symptoms of sciatica caused by disc herniation [13]. In India, the most common age of presentation of diagnosed cases of LDH at a tertiary care hospital is 31 to 40 years (33.33%), and the condition is more common among people from rural areas, those engaged in moderate- to heavy-duty work, and vehicle drivers on poor roads [14]. Therefore, cost-effective and safe medications, as well as nonpharmacological interventions, have the potential to be incorporated into the treatment paradigm if they demonstrate good-quality evidence of effectiveness. This study is an attempt to evaluate the efficacy of *marma* therapy for pain management in patients with LDH-associated radiculopathy. The comparator is physiotherapy, which is a modality advised in the conventional standard of care for the conservative management of this condition [15].

*Marma* points are defined as vital anatomical sites where 2 or more bodily structures—such as muscles, veins, ligaments, bones, or joints—intersect. Etymologically, the Sanskrit term “*marma”* translates to “hidden” or “secret.” The *Sushruta* Samhita classically enumerates 107 such points. The corresponding therapeutic modality, *marma chikitsa,* involves applying targeted pressure to these junctions. This intervention is postulated to facilitate the movement of *prana* (vital energy) through a sophisticated network of subtle channels (*nadis*)*,* ultimately producing symptomatic relief, including analgesia [7]. *Marma chikitsa* is primarily practiced in southern India by traditional medicine healers and schools of martial arts, especially for various acute or chronic musculoskeletal and neuromuscular conditions. However, some practitioners also treat many systemic diseases using *marma chikitsa*, also popularly known as *varmam* in the Tamil language.

If *marma* therapy is demonstrated to be effective and safe in managing pain and enhancing QoL by reducing disability associated with LDH, it may be further evaluated in larger studies to strengthen the evidence base for its effectiveness. Additionally, training a greater number of traditional medicine practitioners in this approach could enable its integration into OPD-level care for patients with LDH. Such simple, noninvasive interventions have the potential to decrease dependence on oral analgesics, such as NSAIDs, among individuals with chronic pain.

## Supplementary material

10.2196/85720 Peer Review Report 1 Peer review report by the Expert Committee for the Development of Draft Integrative Protocols on *Marma Chikitsa*, Central Council for Research in Ayurvedic Sciences, Ministry of Ayush, Government of India.

## Abbreviations

AE: adverse event
CRF: case record form
e-CRF: electronic case record form
IEC: Institutional Ethics Committee
LBP: low-back pain
LDH: lumbar disc herniation
MRI: magnetic resonance imaging
NSAID: nonsteroidal anti-inflammatory drug
ODI: Oswestry Disability Index
OPD: outpatient department
QoL: quality of life
RCT: randomized controlled trial
SF-36: 36-item short form health survey
SLRT: straight leg raise test
VAS: Visual Analog Scale
