# Supplementary material for: Evaluating the Efficacy of Marma Therapy for Pain in Lumbar Disc Herniation With Radiculopathy: Protocol for a Randomized Controlled Trial
Source: JMIR Res Protoc. 2026 May 4;15:e85720. doi: 10.2196/85720 (PMC13138787; doi:10.2196/85720)
Supplement: Peer Review Report 1 [file resprot-v15-e85720-s001.pdf]

protocol draft for Expert vetting

1 message

Drshruti Khanduri <drshruticcras@gmail.com> Fri, Jul 9, 2021 at 12:46
To: Rakesh Narayanan V <drrakeshccras@gmail.com>, Dr. V. K. Lavaniya <vklavaniya@rediffmail.com>
Cc: Bhogavalli CHANDRA SEKHARA RAO <bhogavalli8@gmail.com>, Sophia J <drsophia9754@gmail.com>, vd.psm2008@gmail.com

Please find attached the draft protocol of post stroke rehabilitation and Lumbar Disc Herniation with Radiculopathy and draft letter for experts vetting .

With Regards

Dr. Shruti Khanduri
Research Officer (Ay.),
Central Council for Research in Ayurvedic Sciences(CCRAS),
Ministry of AYUSH, Govt. of India
Room No.108, 1st Floor, JLNBCHA Bhawan
61-65, Institutional Area, Opp. 'D' Block
Janakpuri, New Delhi-110058
Mob.7011166091, 9213121355
E-mail: drshruticcras@gmail.com

---

3 attachments

- **Draft Stroke protocol-08.07.2021.docx**
  176 KB
- **An RCT to evaluate the efficacy of Marma Therapy in Lumbar Disc Herniation with Radiculopathy.docx**
  89 KB
- **Letter to experts- Marma Protocol.docx**
  206 KB

Gmail **Dr. Pallavi Mundada <vd.psm2008@gmail.com>**

Minutes of the meeting of expert Committee for development of drafty integrative protocols on Marmachikitsa on 9th August, 2021 at 11:00 AM through video conferencing-reg.

7 messages

Dr. Narayanam Srikanth <dg-ccras@nic.in> Thu, Aug 12, 2021 at 3:33 PM
To: "vasanthapadma123@gmail.com" <vasanthapadma123@gmail.com>, "madangodbole@yahoo.co.in" <madangodbole@yahoo.co.in>, "pant.mahendra@gmail.com" <pant.mahendra@gmail.com>, "atul_juneja@hotmail.com" <atul_juneja@hotmail.com>, "profpnrao@sdmcahhassan.org" <profpnrao@sdmcahhassan.org>, "dr_bsprasad@rediffmail.com" <dr_bsprasad@rediffmail.com>
Cc: uttarakhandayurved <uttarakhandayurved@gmail.com>, Sumeet Goel <osdt-ayush@gov.in>, Narayanam Srikanth <srikanthccras@gmail.com>, bhogavalli49 <bhogavalli49@gmail.com>, drshruticcras <drshruticcras@gmail.com>, Dr Shruti <drshruti.ayurveda@gmail.com>, drsophia9754 <drsophia9754@gmail.com>, vdpsm2008 <vd.psm2008@gmail.com>, Dr Adarsh <dr.adarshkr25@nic.in>, drpratapmakhija <drpratapmakhija@yahoo.in>, vklavaniya@rediffmail.com, Rakesh Narayan v <drrakeshccras@gmail.com>, "R.K.Rana Stat Officer" <ccras_stat@nic.in>, richa Singhal <richa.singhal2k@gmail.com>, Dr Arunabh <arunabh.tripathi@gov.in>

DRAFT PROTOCOLS - FOR RESTRICTED CIRCULATION

Madam/Sir,

Please find enclosed the attachments on the subject cited above.

Thanks & regards,

(Dr.N.Srikanth)

Director General(Addl.Charge)

केन्द्रीय आयुर्वेदीय विज्ञान अनुसंधान परिषद्

Central Council for Research in Ayurvedic Sciences (CCRAS)

आयुष मंत्रालय, भारत सरकार, Ministry of AYUSH, Govt. of India

न: 61 - 65 सांस्थानिक क्षेत्र, सम्मुख डी ब्लाक, जनकपुरी, नई दिल्ली - 110058

No. 61 - 65, Institutional Area, Opp. 'D'- Block, Janakpuri, New Delhi -110058
Email: ccras_tec@nic.in

संम्पर्क नः / Contact No . 011 - 28522010/28522767

#AYUSH #ZindagiRaheKhush #CCRAS

4 attachments

Minutes of Marma Expert group meeting on 09.08.2021 (1).doc
41K

Annexure III- An RCT to evaluate the efficacy of Marma Therapy in Lumbar Disc Herniation with Radiculopathy- 09.08.2021.docx
101K

Annexure II- Stroke protocol 09.08.21.docx
114K

Minutes of first expert group meeting for finalization of draft protocols on Marmachikitsa.pdf
800K

Dr. Narayanam Srikanth <dg-ccras@nic.in> Thu, Aug 12, 2021 at 3:48 PM
To: vasanthapadma123@gmail.com, madangodbole@yahoo.co.in, pant mahendra <pant.mahendra@gmail.com>, atul juneja <atul_juneja@hotmail.com>, profpnrao@sdmcahhassan.org, dr bsprasad <dr_bsprasad@rediffmail.com>
Cc: uttarakhandayurved <uttarakhandayurved@gmail.com>, Sumeet Goel <osdt-ayush@gov.in>, Narayanam Srikanth <srikanthccras@gmail.com>, bhogavalli49 <bhogavalli49@gmail.com>, drshruticcras <drshruticcras@gmail.com>, Dr Shruti <drshruti.ayurveda@gmail.com>, drsophia9754 <drsophia9754@gmail.com>, vdpsm2008 <vd.psm2008@gmail.com>, Dr Adarsh <dr.adarshkr25@nic.in>, drpratapmakhija <drpratapmakhija@yahoo.in>, vklavaniya@rediffmail.com, Rakesh Narayan v <drrakeshccras@gmail.com>, "R.K.Rana Stat Officer" <ccras_stat@nic.in>, richa Singhal <richa.singhal2k@gmail.com>, Dr Arunabh <arunabh.tripathi@gov.in>

Madam/Sir,

In continuation to the trailing mail, please find enclosed herewith a revised covering letter on the subject cited above.

Kindly ignore the previous covering letter attached to the previous mail.

Thanks

From: "Dr. Narayanam Srikanth" <dg-ccras@nic.in>
To: vasanthapadma123@gmail.com, madangodbole@yahoo.co.in, "pant mahendra" <pant.mahendra@gmail.com>, "atul juneja" <atul_juneja@hotmail.com>, profpnrao@sdmcahhassan.org, "dr bsprasad" <dr_bsprasad@rediffmail.com>
Cc: "uttarakhandayurved" <uttarakhandayurved@gmail.com>, "Sumeet Goel" <osdt-ayush@gov.in>, "Narayanam Srikanth" <srikanthccras@gmail.com>, "bhogavalli49" <bhogavalli49@gmail.com>, "drshruticcras" <drshruticcras@gmail.com>, "Dr Shruti" <drshruti.ayurveda@gmail.com>, "drsophia9754" <drsophia9754@gmail.com>, "vdpsm2008" <vd.psm2008@gmail.com>, "Dr Adarsh" <dr.adarshkr25@nic.in>, "drpratapmakhija" <drpratapmakhija@yahoo.in>, vklavaniya@rediffmail.com, "Rakesh Narayan v" <drrakeshccras@gmail.com>, "R.K.Rana Stat Officer" <ccras_stat@nic.in>, "richa Singhal" <richa.singhal2k@gmail.com>, "Dr Arunabh" <arunabh.tripathi@gov.in>
Sent: Thursday, August 12, 2021 3:33:46 PM
Subject: Minutes of the meeting of expert Committee for development of drafty integrative protocols on Marmachikitsa on 9th August, 2021 at 11:00 AM through video conferencing-reg.
[Quoted text hidden]

Revised covering letter of meeting of Expert Group for finalization of protocols on Marmachakitsa.pdf
374K

Atul Juneja <atul_juneja@hotmail.com> Thu, Aug 12, 2021 at 4:08 PM
To: "Dr. Narayanam Srikanth" <dg-ccras@nic.in>, "vasanthapadma123@gmail.com" <vasanthapadma123@gmail.com>, "madangodbole@yahoo.co.in" <madangodbole@yahoo.co.in>, "pant.mahendra@gmail.com"

<pant.mahendra@gmail.com>, "profpnrao@sdmcahhassan.org" <profpnrao@sdmcahhassan.org>, "dr_bsprasad@rediffmail.com" <dr_bsprasad@rediffmail.com>
Cc: uttarakhandayurved <uttarakhandayurved@gmail.com>, Sumeet Goel <osdt-ayush@gov.in>, Narayanam Srikanth <srikanthccras@gmail.com>, bhogavalli49 <bhogavalli49@gmail.com>, drshruticcras <drshruticcras@gmail.com>, Dr Shruti <drshruti.ayurveda@gmail.com>, drsophia9754 <drsophia9754@gmail.com>, vdpsm2008 <vd.psm2008@gmail.com>, Dr Adarsh <dr.adarshkr25@nic.in>, drpratapmakhija <drpratapmakhija@yahoo.in>, "vklavaniya@rediffmail.com" <vklavaniya@rediffmail.com>, Rakesh Narayan v <drrakeshccras@gmail.com>, "R.K.Rana Stat Officer" <ccras_stat@nic.in>, richa Singhal <richa.singhal2k@gmail.com>, Dr Arunabh <arunabh.tripathi@gov.in>

Dear Sir
Shall appreciate the computational details of sample size with assumptions as discussed
regards
Dr Atul Juneja

From: Dr. Narayanam Srikanth <dg-ccras@nic.in>
Sent: Thursday, August 12, 2021 3:33 PM
To: vasanthapadma123@gmail.com <vasanthapadma123@gmail.com>; madangodbole@yahoo.co.in <madangodbole@yahoo.co.in>; pant.mahendra@gmail.com <pant.mahendra@gmail.com>; atul_juneja@hotmail.com <atul_juneja@hotmail.com>; profpnrao@sdmcahhassan.org <profpnrao@sdmcahhassan.org>; dr_bsprasad@rediffmail.com <dr_bsprasad@rediffmail.com>
Cc: uttarakhandayurved <uttarakhandayurved@gmail.com>; Sumeet Goel <osdt-ayush@gov.in>; Narayanam Srikanth <srikanthccras@gmail.com>; bhogavalli49 <bhogavalli49@gmail.com>; drshruticcras <drshruticcras@gmail.com>; Dr Shruti <drshruti.ayurveda@gmail.com>; drsophia9754 <drsophia9754@gmail.com>; vdpsm2008 <vd.psm2008@gmail.com>; Dr Adarsh <dr.adarshkr25@nic.in>; drpratapmakhija <drpratapmakhija@yahoo.in>; vklavaniya@rediffmail.com <vklavaniya@rediffmail.com>; Rakesh Narayan v <drrakeshccras@gmail.com>; R.K.Rana Stat Officer <ccras_stat@nic.in>; richa Singhal <richa.singhal2k@gmail.com>; Dr Arunabh <arunabh.tripathi@gov.in>
Subject: Minutes of the meeting of expert Committee for development of drafty integrative protocols on Marmachikitsa on 9th August, 2021 at 11:00 AM through video conferencing-reg.

[Quoted text hidden]

Sophia J <drsophia9754@gmail.com> Tue, Aug 17, 2021 at 10:20 AM
To: "Dr. Pallavi Mundada" <vd.psm2008@gmail.com>

---------- Forwarded message ---------
From: **Atul Juneja** <atul_juneja@hotmail.com>
Date: Mon, 16 Aug 2021, 21:44
Subject: Re: Minutes of the meeting of expert Committee for development of drafty integrative protocols on Marmachikitsa on 9th August, 2021 at 11:00 AM through video conferencing-reg.
To: Sophia J <drsophia9754@gmail.com>
Cc: BCS Rao <bhogavalli49@gmail.com>, Drshruti Khanduri <drshruticcras@gmail.com>, Dr. Richa Singhal <richa.singhal2k@gmail.com>

Dear Sir/mam
This is fine how about the other project
regards
Atul Juneja

From: Sophia J <drsophia9754@gmail.com>
Sent: Monday, August 16, 2021 3:25 PM
To: Atul Juneja <atul_juneja@hotmail.com>
Cc: BCS Rao <bhogavalli49@gmail.com>; Drshruti Khanduri <drshruticcras@gmail.com>; Dr. Richa Singhal <richa.singhal2k@gmail.com>
Subject: Re: Minutes of the meeting of expert Committee for development of drafty integrative protocols on Marmachikitsa on 9th August, 2021 at 11:00 AM through video conferencing-reg.

Respected Sir,

As per the discussion held on 09.08.2021, the sample size for the study on stroke has been recalculated based on your suggestion, as follows:

Considering a change of 5 points in Fugl-Meyer Motor Impairment Score at 3 months from Baseline as a clinically meaningful difference, sample size have been calculated as 95 per group

It was assumed that at least 20% participants in physiotherapy group will have a change of >=5 points as compared to 40% participants in the Marma chikitsa group

The sample size has been calculated considering level of significance as 5% and power of the study as 80%.

$\alpha = 0.05$ Hence $Z_{1-\alpha/2} = 1.96$

$1-\beta = 80\%$ hence $Z_{1-\beta} = 0.8416$

$p1 = 0.40$

$p2 = 0.20$

$n = [p1(1-p1) + p2(1-p2)][Z_{1-\alpha/2} + Z_{1-\beta}]^2 / (p1-p2)^2$

$n = 79$

Assuming a dropout rate of 20 %

= 79 + 15.8 = 94.8 (Approximately 95 patients to be included in the trial in each group).Therefore, a total of 190 patients have to be enrolled in the trial.

(*Ref: 1.Wu CY, Huang PC, Chen YT, Lin KC, Yang HW. Effects of mirror therapy on motor and sensory recovery in chronic stroke: a randomized controlled trial. Arch Phys Med Rehabil. 2013 Jun;94(6):1023-30. doi: 10.1016/j.apmr.2013.02.007. Epub 2013 Feb 15. PMID: 23419791.*)&

2. Singh N, Saini M, Kumar N, Srivastava MVP, Kumaran SS, Mehndiratta A. A Case Report: Effect of Robotic Exoskeleton Based Therapy on Neurological and Functional Recovery of a Patient With Chronic Stroke. Front Neurol. 2021;12:680733. Published 2021 Jul 12. doi:10.3389/fneur.2021.680733)

You are kindly requested to give your valuable inputs on this sample size.

Thanking You,

Dr Sophia Jameela
Research Officer (Ay)
Central Council for Research in Ayurvedic Sciences
Jawaharlal Nehru Bharatiya Chikitsa Evam Homoeopathy Anusandhan Bhawan
No 61-65,Institutional Area,*Opp D block, Janakpuri, New Delhi -110058*

[Quoted text hidden]

Dr. Pallavi Mundada <vd.psm2008@gmail.com> Tue, Aug 17, 2021 at 5:00 PM
To: Atul Juneja <atul_juneja@hotmail.com>
Cc: "Dr. Narayanam Srikanth" <dg-ccras@nic.in>, "vasanthapadma123@gmail.com" <vasanthapadma123@gmail.com>, "madangodbole@yahoo.co.in" <madangodbole@yahoo.co.in>, "pant.mahendra@gmail.com" <pant.mahendra@gmail.com>, "profpnrao@sdmcahhassan.org" <profpnrao@sdmcahhassan.org>, "dr_bsprasad@rediffmail.com" <dr_bsprasad@rediffmail.com>, uttarakhandayurved <uttarakhandayurved@gmail.com>, Sumeet Goel <osdt-ayush@gov.in>, Narayanam Srikanth <srikanthccras@gmail.com>, bhogavalli49 <bhogavalli49@gmail.com>, drshruticcras <drshruticcras@gmail.com>, Dr Shruti <drshruti.ayurveda@gmail.com>, drsophia9754 <drsophia9754@gmail.com>, Dr Adarsh <dr.adarshkr25@nic.in>, drpratapmakhija <drpratapmakhija@yahoo.in>, "vklavaniya@rediffmail.com" <vklavaniya@rediffmail.com>, Rakesh Narayan v <drrakeshccras@gmail.com>, "R.K.Rana Stat Officer" <ccras_stat@nic.in>, richa Singhal <richa.singhal2k@gmail.com>, Dr Arunabh <arunabh.tripathi@gov.in>

The sample size for the study is calculated assuming that post treatment mean VAS score of pain in Group-1 will be 2.2 (± 1.3) and in Group 2 mean will be 3.0 (± 1.3). With 95% Confidence Level and 80% power and expecting a dropout rate of 20%, the number of participants to be enrolled in the study should be 49 (approximately 50) in each group. Hence, a total of 100 participants will be enrolled in the trial.Therefore, a total of 100 patients have to be enrolled in the trial.

$n_1 = n_2 = 2\,\sigma^2\,[(Z_{1-\alpha/2}) + (Z_\beta)]^2 \div \Delta^2$

$n_1 = n_2 = 2*(1.3)^2\,[(1.96+0.84)]^2 \div (0.8)^2$

$n_1 = n_2 = 41$

$n = 2n_1 = 2n_2 = 82$

$\Delta = |\mu_2 - \mu_1|$ = absolute difference between two means
σ_1, σ_2 = variance of mean #1 and #2
n_1 = sample size for group #1
n_2 = sample size for group #2
α = probability of type I error (usually 0.05)
β = probability of type II error (usually 0.2)
z = critical Z value for a given α or β
k = ratio of sample size for group #2 to group #1

Reference -

Şahin N, Karahan AY, Albayrak İ. Effectiveness of physical therapy and exercise on pain and functional status in patients with chronic low back pain: a randomized-controlled trial. *Turk J Phys Med Rehabil*. 2017;64(1):52-58. Published 2017 Aug 9. doi: 10.5606/tftrd.2018.1238
Syal Kumar, Thomas Rampp, Christian Kessler, Michael Jeitler, Gustav J. Dobos, Rainer Lüdtke, Larissa Meier, and Andreas Michalsen.The Journal of Alternative and Complementary Medicine.Feb 2017.109-115.http://doi.org/10.1089/acm.2015.0272

You are requested to kindly give your valuable inputs on this sample size.

सादर धन्यवाद,
डॉ. पल्लवी मुंदड़ा
अनुसन्धान अधिकारी (आयु.)
केंद्रीय आयुर्वेद विज्ञान अनुसन्धान परिषद,
आयुष मंत्रालय, भारत सरकार

Gratitude with regards,
Dr. Pallavi Mundada,
Research Officer (Ay.),
Central Council for Research in Ayurvedic Sciences,
Under Ministry of AYUSH, Govt. of India
Mob. 8956606123
https://orcid.org/0000-0003-3326-2165

On Thu, Aug 12, 2021 at 4:08 PM Atul Juneja <atul_juneja@hotmail.com> wrote:
[Quoted text hidden]

A Randomized Controlled Trial to evaluate the efficacy of Marma Therapy in pain management in Lumbar Disc Herniation with Radiculopathy.pdf
1057K

Atul Juneja <atul_juneja@hotmail.com> Tue, Aug 17, 2021 at 6:55 PM
To: "Dr. Pallavi Mundada" <vd.psm2008@gmail.com>
Cc: "Dr. Narayanam Srikanth" <dg-ccras@nic.in>, "vasanthapadma123@gmail.com" <vasanthapadma123@gmail.com>, "madangodbole@yahoo.co.in" <madangodbole@yahoo.co.in>, "pant.mahendra@gmail.com" <pant.mahendra@gmail.com>, "profpnrao@sdmcahhassan.org" <profpnrao@sdmcahhassan.org>, "dr_bsprasad@rediffmail.com" <dr_bsprasad@rediffmail.com>, uttarakhandayurved <uttarakhandayurved@gmail.com>, Sumeet Goel <osdt-ayush@gov.in>, Narayanam Srikanth <srikanthccras@gmail.com>, bhogavalli49 <bhogavalli49@gmail.com>, drshruticcras <drshruticcras@gmail.com>, Dr Shruti <drshruti.ayurveda@gmail.com>, drsophia9754 <drsophia9754@gmail.com>, Dr Adarsh <dr.adarshkr25@nic.in>, drpratapmakhija <drpratapmakhija@yahoo.in>, "vklavaniya@rediffmail.com" <vklavaniya@rediffmail.com>, Rakesh Narayan v

<drrakeshccras@gmail.com>, "R.K.Rana Stat Officer" <ccras_stat@nic.in>, richa Singhal <richa.singhal2k@gmail.com>, Dr Arunabh <arunabh.tripathi@gov.in>

Dear Sir/Mam
If I have understood correctly, we are ignoring the initial pain score which is perhaps could be for the reason that average pain score in two groups is same which gets reduced on an average to 2.2 in interventional group and to 3 units in control group. This assumption can hold only when two groups are similar in terms of pain score -logistically appears difficult, clinicians can clarify better.
ANother general comment on assumptions relating to sample size computation
When we assume some value based experience/literature usually it is not expressed in fractions for instance pain score of 2.2, since this is an assumption and not a hard core figure. hence this can be put as 2 vs 3 instead of 2.3 or if you still wish to retain the close margin may take this assumption as 2.5 (this will take care even if change is 2.3)-might marginally increase sample size
If you are OK with the assumptions initially considered may continue as it is
This is just a humble suggestion
Regards

Atul Juneja

From: Dr. Pallavi Mundada <vd.psm2008@gmail.com>
Sent: Tuesday, August 17, 2021 5:00 PM
To: Atul Juneja <atul_juneja@hotmail.com>
Cc: Dr. Narayanam Srikanth <dg-ccras@nic.in>; vasanthapadma123@gmail.com <vasanthapadma123@gmail.com>; madangodbole@yahoo.co.in <madangodbole@yahoo.co.in>; pant.mahendra@gmail.com <pant.mahendra@gmail.com>; profpnrao@sdmcahhassan.org <profpnrao@sdmcahhassan.org>; dr_bsprasad@rediffmail.com <dr_bsprasad@rediffmail.com>; uttarakhandayurved <uttarakhandayurved@gmail.com>; Sumeet Goel <osdt-ayush@gov.in>; Narayanam Srikanth <srikanthccras@gmail.com>; bhogavalli49 <bhogavalli49@gmail.com>; drshruticcras <drshruticcras@gmail.com>; Dr Shruti <drshruti.ayurveda@gmail.com>; drsophia9754 <drsophia9754@gmail.com>; Dr Adarsh <dr.adarshkr25@nic.in>; drpratapmakhija <drpratapmakhija@yahoo.in>; vklavaniya@rediffmail.com <vklavaniya@rediffmail.com>; Rakesh Narayan v <drrakeshccras@gmail.com>; R.K.Rana Stat Officer <ccras_stat@nic.in>; richa Singhal <richa.singhal2k@gmail.com>; Dr Arunabh <arunabh.tripathi@gov.in>
Subject: Re: Minutes of the meeting of expert Committee for development of drafty integrative protocols on Marmachikitsa on 9th August, 2021 at 11:00 AM through video conferencing-reg.

[Quoted text hidden]

Dr. Pallavi Mundada <vd.psm2008@gmail.com> Wed, Aug 18, 2021 at 3:16 PM
To: Atul Juneja <atul_juneja@hotmail.com>
Cc: Rakesh Narayan v <drrakeshccras@gmail.com>, "R.K.Rana Stat Officer" <ccras_stat@nic.in>, "Dr. Shruti Khanduri" <drshruticcras@gmail.com>, BCS Rao <bhogavalli49@gmail.com>, Dr Shruti <drshruti.ayurveda@gmail.com>

Respected Sir,
The sample size calculation has been revised as per your suggestion. Following is the description of calculation.

According to the previous literature an improvement of 1.7 points in VAS score for pain was reported by physiotherapy. In this study, we anticipate an improvement of at least 1 point more by Marma therapy which is considered minimally of clinical significance (i.e an improvement of at least 2.7 points). Therefore, the sample size of 36 in each group has been calculated with a standard deviation of 1.5 points, $\alpha = 0.05$ and power = 80% as per the below mentioned formula. Adding a drop out rate of 25% the sample size becomes 45 per group. Hence, a total of 90 participants will be included in the trial.

$$n_1 = n_2 = 2\,\sigma^2\,[(Z_{1-\alpha/2}) + (Z_\beta)]^2 \div \Delta^2$$

$n_1 = n_2 = 2*(1.5)^2 [(1.96+0.84)]^2 \div (1.0)^2$

$n_1 = n_2 = 36$

Adding a drop out rate of 25%, the sample size per group becomes 45.

$\Delta = |\mu_2 - \mu_1|$ = absolute difference between two means
σ_1, σ_2 = variance of mean #1 and #2
n_1 = sample size for group #1
n_2 = sample size for group #2
α = probability of type I error (usually 0.05)
β = probability of type II error (usually 0.2)
z = critical Z value for a given α or β
k = ratio of sample size for group #2 to group #1

Reference -

Şahin N, Karahan AY, Albayrak İ. Effectiveness of physical therapy and exercise on pain and functional status in patients with chronic low back pain: a randomized-controlled trial. *Turk J Phys Med Rehabil*. 2017;64(1):52-58. Published 2017 Aug 9. doi: 10.5606/tftrd.2018.1238
Syal Kumar, Thomas Rampp, Christian Kessler, Michael Jeitler, Gustav J. Dobos, Rainer Lüdtke, Larissa Meier, and Andreas Michalsen.The Journal of Alternative and Complementary Medicine.Feb 2017.109-115.http://doi.org/10.1089/acm.2015.0272

Thank you for your valuable suggestions sir.

सादर धन्यवाद,
डॉ. पल्लवी मुंदड़ा
अनुसन्धान अधिकारी (आयु.)
केंद्रीय आयुर्वेद विज्ञान अनुसन्धान परिषद्,
आयुष मंत्रालय, भारत सरकार

Gratitude with regards,
Dr. Pallavi Mundada,
Research Officer (Ay.),
Central Council for Research in Ayurvedic Sciences,
Under Ministry of AYUSH, Govt. of India
Mob. 8956606123
https://orcid.org/0000-0003-3326-2165

[Quoted text hidden]

CENTRAL COUNCIL FOR RESEARCH IN AYURVEDIC SCIENCES

Minutes of the First Expert Group Meeting held on 09.08.2021 at 11:00 AM

A meeting of Expert Committee for review and finalization of clinical trial protocols on efficacy of Marma therapy on select disease conditions (Post-stroke rehabilitation & Lumbar radiculopathy) for Marma therapy was held through VC on 09.08.2021 at 11:00 AM**.** The list of expert group members and CCRAS officers who attended the meeting are at Annexure I

At the outset, Convenor, **Dr. B.C.S Rao**, AD (Ay), Assistant Director (Ay), CCRAS Hqrs welcomed the committee members and gave a brief appraisal of the draft protocols.

The protocols titled "A Randomized controlled trial to evaluate the efficacy of Marma Therapy in Lumbar Disc Herniation with Radiculopathy" and "A randomized controlled trial to evaluate the efficacy of Marma therapeutic regime over physiotherapy on motor, cognitive and quality of life assessment parameters during post-stroke rehabilitation" were presented by the officers from the Council and elaborate discussions ensued on various aspects.
After the detailed deliberation, the Committee suggested the following:-

1) The Sample size for the post stroke rehabilitation trial may be re-approximated taking into account, the difference in the proposed outcome, viz., the improvement in Fugl Meyer Assessment that the randomised controlled trial is designed to reliably detect and could be clinically deemed as an improvement.

2) The committee also proposed to involve a physiotherapist as an investigator in both the trials as physiotherapy regimen is the control arm

3) The title of both the trials may be modified by adding Randomized Controlled Clinical Trial in place of Randomized Controlled trial.

4) For the self-care rehabilitative care regime at home, following the first 30 days, participant adherence may be ensured through telephonic follow-ups

5) In the stroke protocol, the feasibility of conducting CD-4+, CD-8+, and Forkhead box P3 (FoxP3)–positive regulatory T cells (Tregs) markers evaluation may be explored to assess the damage in brain following stroke.

6) Inclusion criteria for the Lumbar Disc Herniation proposal may be revised (only painful condition without motor deficit should be explicitly stated).

7) It was suggested that two independent monitors may be involved to ensure that the trial is in compliance with the approved study protocol.

8) Clarification about the concomitant medications allowed during the trial is necessary. If the participant is already taking any allopathic or any other medications for Lumbar Disc Herniation, a washout period for such ongoing treatment in both the groups may be proposed. Rescue medications like NSAIDs or non NSAIDs needed for pain management during the study period should be recorded.

   The protocols modified after incorporating the suggestions/inputs of the Expert Committee members are annexed at Annexure II & III.

Annexure I

List of Partcipants:

Committee Members who attended the meeting

1. **Prof. M.V.Padma Srivastava**, Professor and Head, Dept of Neurology, Neurosciences Centre, AIIMS, New Delhi
2. **Prof.Madan Godbole**, Department of Endocrinology, Sanjay Gandhi Post Graduate Institute of Medical Sciences, Lucknow, Uttar Pradesh
3. **Dr.B.S.Prasad,** National Commission for Indian System of Medicine, New Delhi
4. **Dr. Atul Juneja**, Scientist E, National Institute of Medical Statistics, Indian Council for Medical Research, New Delhi
5. **Dr. Prasanna N. Rao**, Principal, Sri Dharmasthala Manjunatheswara College of Ayurveda, Hassan, Karnataka
6. **Dr. B.C.S. Rao**, Assistant Director (Ay), Convenor, CCRAS, Hqrs

Member who could not attend the meeting:

Prof. Mahendra Kumar Pant, Professor and Head of Anatomy, Govt Doon Medical College, Dehradun, Uttarakhand

Principal Investigator

Prof.S.K. Joshi, Vice Chancellor Uttarakhand Ayurved University, Dehradun

Officials from CCRAS Headquarters:

1. **Dr. Shruti Khanduri**, RO(Ay), CCRAS, Hdqrs
2. **Dr. V. K. Lavaniya**, RO(Ay), CCRAS, Hdqrs
3. **Dr. Pallavi Mundada**, R.O(Ay), CCRAS, Hdqrs
4. **Dr. Sophia Jameela**, R.O (Ay), CCRAS, Hdqrs
5. **Dr. Rakesh Narayanan**, R.O (Ay), CCRAS, Hdqrs

केन्द्रीय आयुर्वेदीय विज्ञान अनुसंधान परिषद्

आयुष मन्त्रालय, भारत सरकार
जवाहर लाल नेहरू भारतीय चिकित्सा एवं होम्योपैथी अनुसंधान भवन
नं. ६१-६५, इन्स्टिट्यूशनल एरिया, सम्मुख 'डी' ब्लाक, जनकपुरी, नई दिल्ली-११००५८

CENTRAL COUNCIL FOR RESEARCH IN AYURVEDIC SCIENCES

Ministry of AYUSH, Govt. of India
Jawahar Lal Nehru Bhartiya Chikitsa Evam Homoeopathy Anusandhan Bhawan
No. 61-65, Institutional Area, Opp. 'D' Block, Janakpuri, New Delhi-110058

Gram : **"AYUSH"**
Fax : 28520748
EPBX
28525852, 28520501
28522524, 28525831
28525862, 28525883
28525897

फा.सं: 3-37/2021-सीसीआरएएस/प्रशा./ 2309

दिनांक:12.08.2021

12 AUG 2021

सेवा में,

संलग्न सूची के अनुसार ।

विषय: दिनांक 09.08.2021 को प्रात: 11:00 बजे मर्मचिकित्सा के प्रोटोकॉल को अंतिम रूप देने पर आयोजित प्रथम विशेषज्ञ समूह की बैठक के कार्यवृत्त के संदर्भ में।

महोदय/महोदया,

उपरोक्त विषय संबंधित आयोजित प्रथम विशेषज्ञ समूह की बैठक के कार्यवृत्त आपकी सूचना हेतु संलग्नक है।

भवदीय

दिलीप 12/08/2021

(दिलीप जैन)

प्रशासनिक अधिकारी (परियोजना एवं सर्तकता)
कृते महानिदेशक अतिरिक्त प्रभार

संलग्न: यथोपरी

A Randomized Controlled Trial to evaluate the efficacy of Marma Therapy in pain management in Lumbar Disc Herniation with Radiculopathy

Protocol Version V.1 10.08 2021

Clinical Trial Protocol

Central Council for Research in Ayurvedic Sciences

Ministry of AYUSH, Government of India

61- 65, Institutional Area, Opposite "D" Block, Janakpuri

New Delhi - 110058

CENTRAL COUNCIL FOR RESEARCH IN AYURVEDIC SCIENCES
CLINICAL TRIAL PROTOCOL

1) **Title of the study**: A Randomized Controlled Trial to evaluate the efficacy of Marma Therapy in pain management in Lumbar Disc Herniation with Radiculopathy

2) **Participating Centre**: Uttarakhand Ayurveda University, Dehradun, Uttarakhand

3) **Name and address of the sponsor**:

   Central Council for Research in Ayurvedic Sciences (C.C.R.A.S),
   Jawaharlal Nehru Bhartiya Chikitsa Evam Homoeopathy Anusandhan Bhawan
   61-65, Institutional Area, Opposite D-Block,
   Janakpuri, New Delhi – 110058

4) **Particulars of the Investigator(s):**

**Sr. No**	**Name of the Institute**	**Name of the Principal Investigator**	**Name of the Co-Investigator**

5) **OBJECTIVES**

- **Primary Objective**:
  a) To evaluate the efficacy of Marma therapy in reducing pain due to Lumbar Radiculopathy attributed to Intervertebral Disc Herniation
- **Secondary Objective:**
  a) To evaluate the role of Marma therapy in improving Quality of Life of patients with Lumbar Radiculopathy attributed to Intervertebral Disc Herniation
  b) To evaluate the effect of Marma therapy on functional disability among patients with Lumbar Radiculopathy attributed to Intervertebral Disc Herniation
  c) To assess the safety of Marma therapy in patients with Lumbar Radiculopathy due to Intervertebral Disc Herniation

6) METHOD:-

**a)** Study Type	:	Interventional
**b)** Purpose	:	Treatment
**c)** Masking	:	Open label
**d)** Control	:	Controlled
**e)** Timing	:	Prospective
**f)** End Point	:	Efficacy
**g)** No. of Groups	:	Two
**h)** Sample size	:	100 (50 in each group)

i) Timelines:-

- Total study period : **12 months**
- Trial Preparation (Staff recruitment & Medicine procurement): 01 month
- Recruitment : 07 months
- Treatment period : 04 weeks
- Follow-up (post treatment) : 08 weeks
- Statistical Analysis and report writing : 01 month

j) Eligibility Criteria:-

1] Inclusion Criteria

1. Participants of any gender in the age group 30-60 years
2. Participants diagnosed with Lumbar Radiculopathy with unilateral radiating pain (either left or right lower limb), due to Intervertebral disc herniation confirmed by MRI (disc herniation between L3 and S1 level; at single or double level) provided no neurologic deficit is evident.
3. Positive straight leg raise test (ipsilateral or contralateral pain in leg, buttock or back at 70 degrees or less of leg elevation and the pain is typically worsened by dorsi-flexion of ankle or neck flexion after slowly lowering the leg until the patient no longer feels pain)
4. Willingness to provide informed consent and adhere to the study protocol for a period of 3 months

2] Exclusion criteria

1. Indication for surgical intervention for disc herniation like severe motor deficit (motor power of lower limbs assessed through Medical Research Council Manual Muscle Testing scale ≤3), severe spinal stenosis; excruciating pain that cannot be managed by conservative treatment, foraminal stenosis, conjoint nerve root, perineural cyst etc.
2. Patients who have received non pharmacological interventions like physiotherapy, traction, manual therapy, etc. for the management of lumbar disc herniation in last 3 months.
3. History of or evidence of any of the following - Osteoporotic lumbar fracture, presence of inflammatory or infective diseases that affect spinal morphology, such as ankylosing spondylitis, Spondylodiscitis or inflammatory spondylitis, spondylolisthesis, Pott's spine, Pyriformis syndrome, sacro-iliitis
4. History of spinal surgery in last 2 years or having epidural fibrosis.
5. Patients with caudaequina syndrome or neurological deficits such as foot drop, limb muscle wasting and bowel/bladder incontinence or Non-ambulatory patients with Monoplegia/ Paraplegia/ Hemiplegia
6. Evidence or history of spinal trauma or Spinal Malignancy
7. Presence of other medical condition presenting with numbness and pain in lower extremities, such as diabetic polyneuropathy and peripheral vascular disease, Motor Neuron Disease, Multiple Sclerosis, Stroke or Cognitive Impairment.
8. Major coexisting medical condition such as cancer, chronic obstructive pulmonary disease, cardiovascular disease and severe hepatic and renal dysfunction.
9. Obesity (BMI greater than or equal to 30 kg/m^2)
10. Metallic implants like pace makers, hearing aid implants etc. and other contraindications for MRI
11. Any other condition that as per the investigator, is contraindicated for the intervention in the study.

*Note:- If the patients are taking any internal or external ongoing medications for management of pain or disc herniation, they will be instructed to stop such treatment. Washout period of 1 week will be given before baseline evaluation, if the patient is currently taking any such treatment.

7) Interventions:-

Group I: Marma Therapy + Trayodasanga Guggulu

A] Marma Therapy -

- **Description of therapy**: Stimulation of *Kshipra, Gulpha, Indrabasti, Katikataruna, Janu,* marmas of affected limb/ both sides)
- **Duration of therapy**: 04 weeks
- **Number of sessions/day**: 03
  (sessions may be done between 5 to7 am, 12 to 2 pm and 5 to 7 pm)
- **Duration of each session**- Each session would last 1-2 minutes (Each stimuli would last 0.8 sec)

Marma description:

1. **Katikataruna**: Both sides of the vertebral column over the hip bone
2. **Kshipra:** Point located between the great toe and second digit (Lower limb)
3. **Janu-** Posterior aspect of the knee joint
4. **Indravasti-** 8 Finger breadth above the first skin fold of the ankle joint
5. **Gulpha-** Just posterior to the medial malleolus

Description of Marma procedure:

- Participant would be advised to wear loose and comfortable clothing (without tie, belt, stocking and such binding clothes)
- Marma stimulation would be done on the participant in lying down position (prone/supine as per the site to be stimulated)
- Marma points will be stimulated by gentle pressure/vibration using the pulp of thumb/fingers and inner side of the knuckle
- Each session would last 1-2 minutes with each stimulus lasting 0.8 seconds (approx. one cardiac cycle)
- Each marma point would be stimulated in each session and there would be 3 sessions per day
- Marma points would be stimulated from upward to downward direction
- Marma stimulation in female patients will be done first in the left extremity followed by right extremity and vice versa in males

B] TrayodasangaGuggulu (API- Part II, Vol. II; AFI – Part 1)

Dose	2 tablets thrice daily
Dosage form	Tablet of 500 mg each
Route of Administration	Oral
Time of Administration	Thrice a day 2 hours after meals
Anupana	Lukewarm water
Duration of therapy	12 weeks

Group II: Physiotherapy + Trayodasanga Guggulu

A] Physiotherapy –

- **Description of therapy**: Electrotherapy, Therapeutic Exercise and Manual Therapy Session
- **Duration of therapy**: 30 minutes for Four weeks
- **Number of sessions/week:** 05

1.) <u>Electrotherapy</u>

TENS - Frequency of 20 Hz; pulse width of 100 µs; continuous mode and two channels for 15 minutes. Electrodes can be placed on the lines of the T12 and S1 vertebra and intensity can be increased slowly to tolerable painful limits.

2.) Hot pack- In water with a temperature of 54°C, for at least 15 minutes, placed at low back with the patient in Hook-lying position, for comfort and pain reduction.

3.) <u>Therapeutic exercise (As per applicability case to case)</u>

A) **Drawing In Maneuver (Abdominal Hollowing Exercise** for Transverse Abdominis Activation) -

Patient position - Hook-lying (with knees 70˚ to 90˚ and feet resting on an exercise mat)

Procedure - The patient will be instructed to breathe in, breath out, then gently draw the belly button in, toward the spine to hollow out the abdominal region and to try to maintain the position for 10 seconds

B) **Multifidus Activation and Training -**

Patient position- Prone or side-lying

Procedure- Palpating digits (thumbs or index fingers) will be placed immediately lateral to the spinous processes of the lumbar spine and the patient will be instructed to "swell the muscle" out against the palpating digits then to draw in and hold 10 seconds.

C) **Cat-camel exercise:-**

Patient position- quadruped position

D) **Arm lifts and bracing and leg lifts and bracing**

Patient position- quadruped position

E) **Single knee to chest:-**

Patient position- Supine lying

Procedure- Try to bring one knee with other leg straight towards the chest.

Progression- After a week, the ball can be placed under the lower leg and later the exercise is performed in the same position with the legs in the air.

F) **Exercises to promote mobility, strength and range of motion in low back** –

Pelvic tilts, Straight leg raise test, supine bridging and Side lying hip abductor exercise. The patient will be instructed to perform 1 set of 10 repetitions of each exercise in a day.

G) **Flexibility training:-**

Tailor made stretching exercise will be given to patient to improve flexibility for the hamstrings, piriformis, and Gastrocnemius and Tensor fascia lata.

H) **Educational training-**

Patient will be advised to avoid sitting for a long periods of time, avoid forward bending, lift heavy object from ground using leg with knee bending and try to tighten the stomach muscles.

B] Trayodasanga Guggulu (API- Part II, Vol. II; AFI – Part 1)

Dose	2 tablets thrice daily
Dosage form	Tablet of 500 mg each
Route of Administration	Oral
Time of Administration	Thrice a day 2 hours after meals
Anupana	Lukewarm water
Duration of therapy	12 weeks

OUTCOMES

Primary Outcomes:-

1) Reduction in the intensity of pain in lumbo-sacral region and legs (radiating pain) perceived by the patients suffering from lumbar radiculopathy due to lumbar disc herniation.

Secondary Outcomes:-

1) Improvement in functional disability index score
2) Change in neurodynamic evaluation through Straight Leg Raise test
3) Improvement in Quality of Life
4) Safety of Marma therapy in patients with lumbar disc herniation
5) Any change in the grade of disc herniation evident in MRI
6) Decreased dependence on allopathic analgesics
7) Compliance of patients to marma therapy

8) OUTCOME MEAUSRES:-

- **Primary outcome measures:**

1) Change in Visual Analogue Scale 10 cm (VAS) score*

[**Time frame of assessment:** at baseline, every week till 4 weeks, biweekly till 12th week]

** The VAS is a point scale measuring 100 mm where the end points are the extremes of no pain and worst pain. The worker is asked to spot the level of his back pain on the scale.The NPRS is a segmented numeric version of the visual analog scale (VAS) in which a respondent selects a whole number (0–10 integers) that best reflects the intensity of his/her pain. Here, 10 cm scale will be used to assess the pain intensity perceived by the patient.*

- **Secondary Outcome measures**:

1) Change in Oswestry Disability Index ***Version 2.0*** (ODI)#

   [**Time frame of assessment:** at baseline, at the end of 2 weeks, 4 weeks, 8 weeks and 12 weeks]

2) Short form 36 (SF-36)* [**Time frame of assessment:** at baseline, at the end of 2 weeks, 4 weeks, 8 weeks and 12 weeks]

3) Straight leg raise test (SLR)$

   [**Time frame of assessment:** at baseline, every week till 4 weeks, biweekly till 12th week]

4) Adverse Events reported during the treatment period [**Time frame of assessment:** At every visit]

5) Frequency of use of rescue medicines for pain management during the treatment period [**Time frame of assessment**: At every visit]

6) Grade of disc herniation evident in MRI lumbo-sacral spine [**Time frame:** at baseline, at the end of 12 weeks]

7) Proportion of participants who completed the study as per protocol

# Oswestry Disability Index (ODI): *The ODI has been developed to assess pain related disability in people with acute, subacute, or chronic low back pain. It consists of 10 multiple-choice questions of LBP one item on pain and nine items on daily activities (walking, sitting, lifting, personal care, standing, sex life, sleeping, social life, and travelling), and for every question the patient chooses one answer out of six that best express his/her disability. The test is considered the 'gold standard' of low back functional outcome tool.* ***Score interpretation:*** *ODI score ranges from 0% (no disability) to 100% (maximum disability). Scores from 0% to 20% point to minimal disability, 21% to 40% point to moderate disability, 41% to 60% point to severe disability, 61% to 80% point to crippled or homebound, and 81% to 100% point to bedridden*

$*The straight leg raise test (SLR) also called the Lasegue test, is a fundamental neurological maneuver during the physical examination of the patient with lower back pain aimed to assess the sciatic compromise due to lumbosacral nerve root irritation. The SLR test is called as positive when there is a sensation of pain by applying pressure to the Piriformis muscle and his tendon, particularly when the hip is flexed at an angle of 90 degrees and the knee is extended. The pain or tenderness is often localized in the vicinity of the greater sciatic notch.*

**As part of the Medical Outcomes Study (MOS), a multi-year, multi-site study to explain variations in patient outcomes, RAND developed the 36-Item Short Form Health Survey (SF-36). This 36-Item Short Form Health Survey questionnaire (SF-36)is a very popular instrument for evaluating Health-Related Quality of Life. It measures eight scales: physical functioning (PF), role physical (RP), bodily pain (BP), general health (GH), vitality (VT), social functioning (SF), role emotional (RE), and mental health (MH). Component analyses showed that there are two distinct concepts measured by the SF-36: a physical dimension, represented by the Physical Component Summary (PCS), and a mental dimension, represented by the Mental Component Summary (MCS).*

The patients will be categorized on the basis of history of onset of symptoms as Acute, Sub-Acute and chronic cases in both groups and the data of efficacy will be compared accordingly.

9) TIMELINE OF ASSESSMENTS :-

Prior to selection – (Screening)

- Informed Consent
- Eligibility evaluation – Clinical assessment, Radiological and laboratory tests

[Spine examination, neurological assessment, Systemic examination, Straight Leg Raise Test **(**SLR), Magnetic Resonance Imaging (MRI) of Lumbo-sacral spine, Laboratory investigations (Routine hematological and biochemistry tests, urine test)]

If the patients are taking any internal or external ongoing medications for management of pain or disc herniation, they will be instructed to stop such treatment. Washout period of 1 week will be given before baseline evaluation, if the patient is currently taking any such treatment.

At Baseline

- General information (Personal Identification and Demographic profile)
- Medical history, General Physical and Systemic examination
- Complete general and neurological examination
- Assessment of pain by 10 cm VAS scale
- Assessment of functional disability by Oswestry Disability Index *Version 2.0* (ODI) **(Annexure 1)**
- Assessment of Quality of life through SF-36 **(Annexure 2)**
- Record of NSAIDs/analgesic medications used during the last 14 days
- Assessment of Ayurvedic Parameters
- Instructions about the Dos and Donts as per standard of care in the said condition.

- Allocation in either group - Participants will be randomly allocated to either Marma therapeutic regime arm or physiotherapy arm as per the sealed envolopes received from the co-coordinating centre or the funding agency, where randomisation will be done centrally by using a computer generated random number sequence.

*Note: The patients will be allowed to continue their medications for comorbidities if any.

Follow-up Visits – Weekly till 4 weeks and biweekly till 12 weeks

- Marma Therapy or Physiotherapy daily till 4 weeks
- Clinical Assessment and assessment of outcome measures as per the timelines.
- Recording need for rescue medications for pain management
- Monitoring incidence of Adverse events
- Instructions about the Dos and Donts as per standard of care in the said condition

At the end of the treatment

- Complete general and neurological examination (to assess the neurological deficits and dermatomal distributions of the sensory level)
- Assessment of Ayurvedic Parameters
- Assessment of pain by 10 cm VAS scale
- Assessment of Disability by Oswestry Disability Index *Version 2.0* (ODI) (**Annexure 1**)
- Assessment of Quality of life through SF-36
- Recording need for rescue medications for pain management
- Monitoring incidence of Adverse events
- Magnetic Resonance Imaging - Lumbar spine

10) Statistical Analysis -

Sample size: The sample size for the study is calculated assuming that post treatment mean VAS score of pain in Group-1 will be 2.2 (± 1.3) and in Group 2 mean will be 3.0 (± 1.3). With 95% Confidence Level and 80% power and expecting a dropout rate of 20%, the number of participants to be enrolled in the study should be 49 (approximately 50) in each group. Hence, a total of 100 participants will be enrolled in the trial.Therefore, a total of 100 patients have to be enrolled in the trial.

$n_1 = n_2 = 2\,\sigma^2\,[(Z_{1-\alpha/2}) + (Z_\beta)]^2 \div \Delta^2$

$n_1 = n_2 = 2*(1.3)^2\,[(1.96+0.84)]^2 \div (0.8)^2$

$n_1 = n_2 = 41$

$n = 2n_1 = 2n_2 = 82$

$\Delta = |\mu_2 - \mu_1|$ = absolute difference between two means

σ_1, σ_2 = variance of mean #1 and #2

n_1 = sample size for group #1

n_2 = sample size for group #2

α = probability of type I error (usually 0.05)

β = probability of type II error (usually 0.2)

z = critical Z value for a given α or β

The above assumptions are based on following studies

1) Şahin N, Karahan AY, Albayrak İ. Effectiveness of physical therapy and exercise on pain and functional status in patients with chronic low back pain: a randomized-controlled trial. *Turk J Phys Med Rehabil*. 2017;64(1):52-58. Published 2017 Aug 9. doi: 10.5606/tftrd.2018.1238
2) Syal Kumar, Thomas Rampp, Christian Kessler, Michael Jeitler, Gustav J. Dobos, Rainer Lüdtke, Larissa Meier, and Andreas Michalsen.The Journal of Alternative and Complementary Medicine.Feb 2017.109-115.http://doi.org/10.1089/acm.2015.0272

Data Analysis: The categorical variables in the study data will be summarized as number (percentage). The continuous data having normal distribution will be represented as mean (SD), and data not following normal distribution as median (min-max). Parametric data will be analyzed by paired t-test and independent sample t-test for within and between group analysis respectively whereas non-parametric data will be calculated by Wilcoxon signed rank test and Mann-Whitney test for within and between group analysis respectively. Other appropriate tests may also be used as per requirement. The level of significance will be 5 %. All the data analysis will be done using the STATA software version 16.1.

The patients will be categorized on the basis of history of onset of symptoms as Acute, Sub-Acute and chronic cases in both groups and the data of efficacy will be analysed and compared accordingly.

Randomization: Statistical package for Social Sciences (SPSS) version 15.0 will be used to generate the random number sequence. The participants will be randomized to two study groups in the ratio of 1:1.

Allocation Concealment: Allocation concealment will be ensured, as the randomisation code will not be released until the patient has been recruited into the trial, i.e., until all baseline measurements have been completed. Sequentially numbered, opaque, sealed envelope (SNOSE) technique will be administered by the trial co-ordination centre.

Blinding: As the study is open-label, blinding of participants will not be done. However, the outcome assessors will not be aware of the allocation of the participant during the study period.

Data collection methods: After informing the participants about the research study as per Patient Information Sheet, written consent (in triplicate) will be taken from the participants before screening them. After screening the participants, the eligible participants will be included in the study and data will be collected in a case record form. The data will be subsequently recorded in an e-format for statistical analysis and record.

Data management: The data will be collected in case record forms. It will be then entered in the Electronic formats. Meticulous data inspection and data cleaning will be done intermittently during the study period. The data collected from all the participants will be kept confidential and safe. After the completion of the study conduction period, the collected data will be anonymised before statistical analysis.

Data Monitoring: The study will be monitored by Data and Safety Monitoring Board (DSMB). An Interim analysis, if required can be done when at least 50% participants have completed their trial period. CCRAS's Central Bio statistical Monitoring Unit (CBMU) and the technical officers involved in project administration will monitor the progress of the trial through regular site visits.

11) **Adverse Event or Adverse Drug Reaction (AE/ADR):** Any adverse event or Adverse Drug Reaction, if observed during treatment period or during follow up visits will be clearly documented and reported to the Ethics committee and the sponsor(s) at the earliest. All efforts to manage the same will be done appropriately and timely.

12) **Concomitant Medication:** Participants enrolled will be instructed to avoid self medication for any ailment and will be clearly instructed to consult the treating investigator for any symptom or complaint or any unusual feeling. The investigator will record any medication(s) he/she may prescribe to alleviate their ailments.

13) **Rescue Medication:** To alleviate any emergency medical condition, the use of any rescue medication will be permitted as per the wisdom/discretion of the Principal Investigator. However, the same will be explicitly documented in the Case Record Form.

14) **Withdrawal Criteria:**

1. Patient not willing to continue or non-compliant with the study procedure.
2. Patient develops life threatening complication or any other severe illness because of other pathology which requires urgent treatment or necessitating the institution of new modalities of treatment during study period.

3. Adverse effect (AE)/ Adverse drug reaction (ADR) necessitating hospitalization.

   **The decision to withdraw a participant from the trial would be taken only by the Investigator, who will then have to provide detailed justification and also indicate the line of further management-if needed. The same needs to be informed to the Sponsor and the Ethics Committee within two working days.

15) **Drop-Outs**: Three to six telephone contacts will be attempted over the course of one week and documented by key research personnel before considering the participant lost to follow-up. An attempt will be made to record the reason for drop outs, if any during the clinical trial.

16) **Ethics**: The trial will be conducted in accordance with ethical principles as per Declaration of Helsinki for biomedical research and ICMR ethical guidelines involving human participants (2017), and that are consistent with Indian / ICH Good Clinical Practice (GCP) guidelines.

Institutional Ethics Committee (IEC): Prior to commencement of the trial, the protocol, the participant information sheet and the consent form will be submitted to the Institutional Ethics Committee and written approval will be obtained from the IEC. Protocol amendments if any will also be approved by the IEC as per usual procedure.

Patient information sheet and Consent form: Possibly eligible patients will be provided verbal and written information about the trial in language that he/she can read and understand. The Investigator will ensure that the participant is fully informed about the aim, procedures, risks and expected benefits of the trial. It will be emphasized that participation is voluntary and participants have the right to opt out of the trial at any time without any prejudice. Then a signed Informed Consent will be obtained from the participants prior to the screening process.

17) **Travel / Incidental support**: A consolidated amount of Rs.100/- per visit during enrollment and then on follow-up day will be given to the patient.

18) **Coordination of Trial**:

Central Council for Research in Ayurvedic Sciences (CCRAS),
Under Ministry of AYUSH, Government of India
Jawahar Lal Nehru Bhartiya Chikitsa Evam Homoeopathy Anusandhan Bhawan,
61-65, Institutional Area, Opposite 'D' Block, Janakpuri, New Delhi-110058, INDIA
